# MetaProx: the database of metagenomic proximons

**DOI:** 10.1093/database/bau097

**Published:** 2014-10-04

**Authors:** Gregory Vey, Trevor C. Charles

**Affiliations:** Department of Biology, University of Waterloo, 200 University Ave. West, Waterloo ON, N2L 3G1, Canada

## Abstract

MetaProx is the database of metagenomic proximons: a searchable repository of proximon objects conceived with two specific goals. The first objective is to accelerate research involving metagenomic functional interactions by providing a database of metagenomic operon candidates. Proximons represent a special subset of directons (series of contiguous co-directional genes) where each member gene is in close proximity to its neighbours with respect to intergenic distance. As a result, proximons represent significant operon candidates where some subset of proximons is the set of true metagenomic operons. Proximons are well suited for the inference of metagenomic functional networks because predicted functional linkages do not rely on homology-dependent information that is frequently unavailable in metagenomic scenarios. The second objective is to explore representations for semistructured biological data that can offer an alternative to the traditional relational database approach. In particular, we use a serialized object implementation and advocate a *Data as Data* policy where the same serialized objects can be used at all levels (database, search tool and saved user file) without conversion or the use of human-readable markups. MetaProx currently includes 4 210 818 proximons consisting of 8 926 993 total member genes.

**Database URL:** http://metaprox.uwaterloo.ca

## Introduction

Currently, much interest exists in the field of computational biology regarding the effective storage, dissemination and harnessing of large data sets. In particular, there is a concern that the current tools and approaches no longer scale up to the present volume of data, thus resulting in a bottleneck in the synthesis of knowledge from data (1). Metagenomic data are no exception to this trend with open-access reads in the Sequence Read Archive (SRA) (2) exceeding 100 Tb by 2011, with metagenomic sequences accounting for 11% of all bases (3). Open-access reads in the SRA now (June 2014) total >1200 Tb. Although the functional annotation and analysis of these data are crucial, the tools currently available to accomplish these tasks have not evolved to match the rate of data generation capabilities (4). Therefore, the development of protocols and tools that can capitalize on the vast availability of metagenomic data represents a major goal for computational biologists.

**Figure 1.**
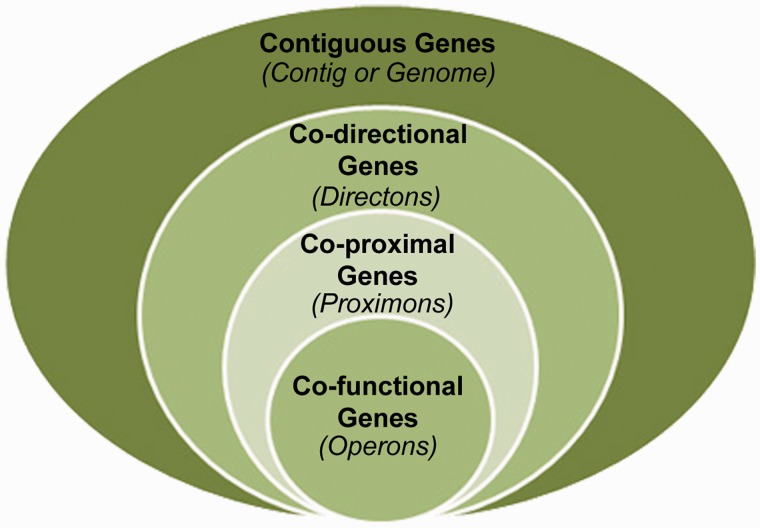
The proximon proposition. The proximon class (co-proximal genes) is shown from a set theoretic perspective as a subset of the directons (co-directional genes) and a superset of the operons (co-functional genes).

The prediction of metagenomic operons offers a means to reveal functional interactions in the absence of knowledge about orthologous relationships (5, 6). Specifically, co-directional intergenic distances can be used to infer operon candidates according to a predicted level of confidence. However, it is at this juncture that some conceptual ambiguity exists. This is because the structures that are identified by this protocol are not guaranteed to be operons, yet they are more significant than the general case of directons (series of contiguous co-directional genes) because their member genes exhibit extreme proximity with respect to adjacent pairwise distances. Therefore, we propose that these structures represent their own unique class situated as a subset of the directon class and a superset of the operon class (see Figure 1), and we propose the term *proximon* to denote a proximally significant directon. The proximon proposition constitutes an essential conceptual demarcation because the prediction of operons on the basis of gene co-direction and proximity is derived from empirical data using *Escherichia coli* K12 (7), and the extensibility of such a model to diverse and/or unique metagenomic taxa is currently unknown (8). Therefore, this distinction is intended to disambiguate proximally significant directons from true operons and to serve as a caveat of the challenges involved in metagenomic operon prediction.

Although established resources already exist with respect to predicted operons from genomic sources (9, 10), we are not aware of any analogous tools that operate at the metagenomic level. Therefore, we have developed MetaProx: the database of metagenomic proximons. MetaProx provides a searchable repository of proximon objects (i.e. operon candidates) conceived with the goal of accelerating research involving metagenomic functional interactions (see Applications). MetaProx currently includes 4 210 818 proximons consisting of 8 926 993 member genes (see Data Generation).

## Implementation

In addition to filling a currently unoccupied informational niche, a secondary goal of MetaProx is to explore representations for semistructured biological data that can offer an alternative to the traditional relational database approach. Although the traditional relational model has been ubiquitously applied with great success, MetaProx has been implemented using a serialized object design motivated primarily by the semistructured and encapsulated nature of biological data, combined with the sparsity of metagenomic functional annotations. While biological data such as annotation records are amenable to representation using relational modeling, the efficacy of such an undertaking is questionable. Beyond the counterintuitive flattening of biological objects composed in turn of other composite objects lurks the tenuous assumption that the fields composing the data are regular and recurrent. In the case of metagenomic functional annotations this assumption does not hold because metagenomic genes exhibit a high degree of variability in the number and type of annotations that they contain (see Table 1). Given the sparsity of the annotations, the relational model has two choices: either offer a vast number of fields to capture all possible combinations of annotation data where most fields will be empty for any given record or offer a limited number of fields to reduce wasted storage where many records will have some of their annotation data omitted (or some combination of these policies). In contrast, an object-based approach to data structuring achieves a verbatim representation by directly storing a variable length list of annotation objects of any type (see Data Model).

**Table 1:** Sparsity of metagenomic functional annotations

Record	COG Cat.	Pfam	TIGRfam	KEGG Mod.	MetaCyc Path.	EC Num.
2000000060	2	2				
2000000140	2	1		2	17	1
2000000300	4	1				
2000000320						
2000000360			1			
2000001710	1	2			3	1

Excerpts from several metagenomic gene annotation records are shown with counts for their respective functional annotations across six different annotation categories. All records were obtained from the Sludge/US Phrap Assembly metagenome, publicly available from the IMG/M: Taxon Object ID 2000000000.

Beyond structuring considerations, our approach to data representation has been inspired by specifications like the common object request broker architecture (CORBA) (11), and we advocate a *Data as Data* policy where the same serialized objects persist across all levels, including the database layer, the application layer and even for the materialization of saved user files. This is in contrast to the lingering perception that biological data should be both transformable and humanly readable, considerations that fuel the ongoing dominance of verbose textual and markup-based representations, like XML or JSON. We believe that data-centric research could be generally accelerated if developers begin to adopt the exchange of serialized objects, rather than maintaining the status quo of inflating computational data into delimited text or markups and then deflating them again during subsequent computation.

### Data model

**Figure 2.**
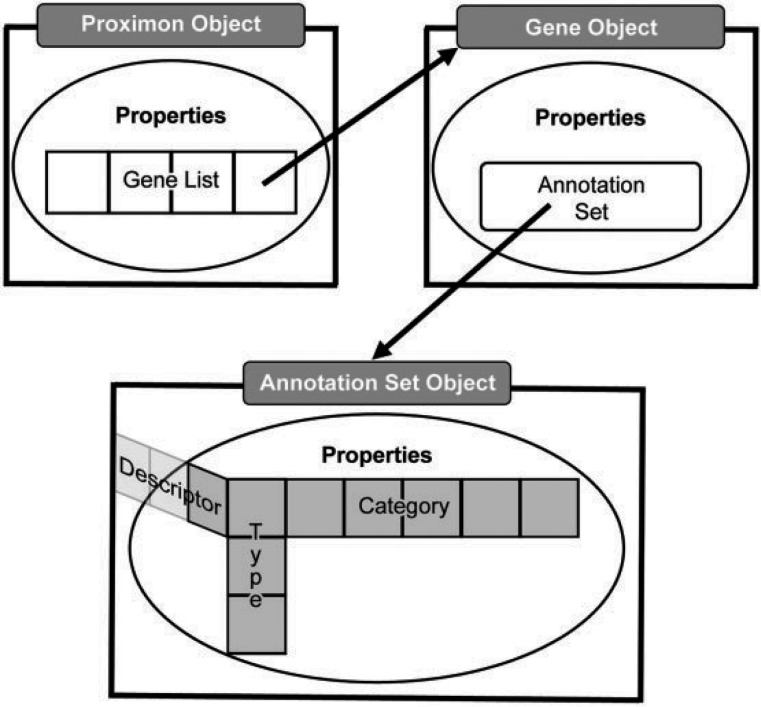
Abstract data model. The top level proximon object is shown with a list of gene objects encapsulated among its properties such that a gene object encapsulates an annotation set object that subsequently encapsulates a 3D list of annotation features, including, type, category and descriptor.

MetaProx uses a hierarchy of Java classes to support the representation and functionality of proximon objects where the manipulation or extraction of data occurs directly by way of a specified application programming interface (API). Given the aforementioned irregularities of a semistructured data, a proximon object is essentially a multidimensional list where dimensionality is constant but the length and contents of a list at any given dimension are highly variable (see Figure 2). At the top level, a proximon object contains a list of gene objects that correspond to its member genes. In turn, each encapsulated gene object contains its own collection of functional annotations in the form of a variable length list of annotation types, each of which contains one or more categorical values and corresponding functional descriptors.

Queries execute by iterating over a subset of proximons where each proximon subsequently iterates over its member genes and in turn each member gene iterates over its particular collection of functional annotations. Specifically, a query object uses the API to perform comparison operations and/or check substring occurrences for each candidate proximon, in a manner similar to the db4o native query (12). Qualifying proximons are added to a sorted results queue and the queue is returned when all proximons are exhausted.

### Data generation

**Figure 3.**
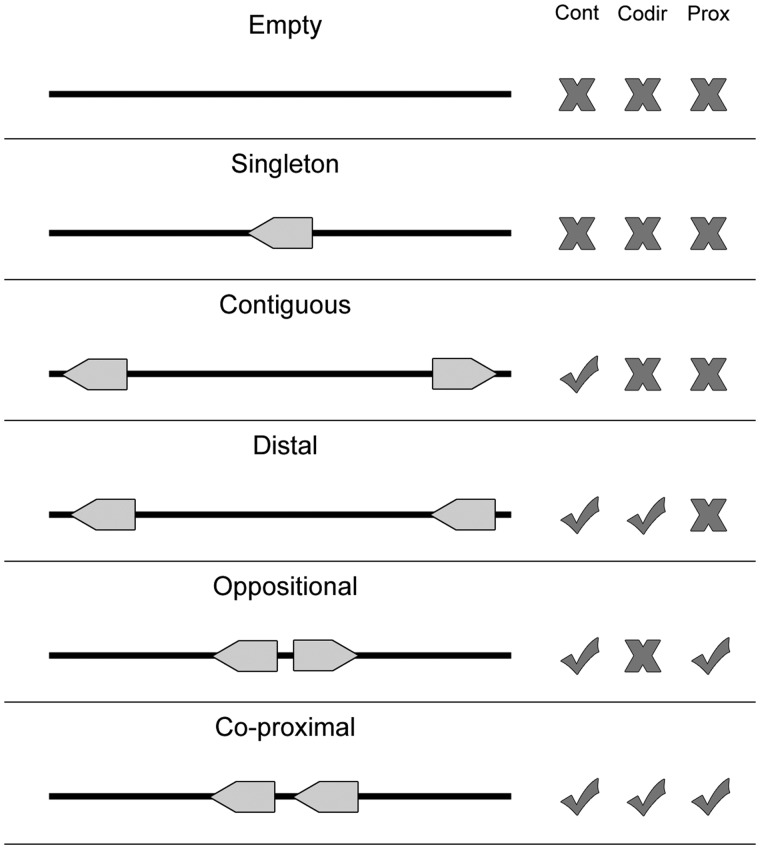
Proximon selection criteria. Various configurations are shown for a metagenomic scaffold that contains zero (Empty), one (Singleton) or two (all other cases) genes. Each configuration is considered with respect to whether it exhibits multiple contiguous genes (Cont), genes that are co-directional (Codir) and genes that are co-proximal (Prox). Only the last configuration meets all the criteria required by the proximon definition.

The proximon data and corresponding metagenomic genes were derived from metagenomic data obtained from the Integrated Microbial Genomes with Microbiome Samples metagenomics database (IMG/M) (13). Specifically, proximons were generated from available metagenomic gene coordinates using a previously published method (5, 6, 8) for identifying metagenomic operon candidates based on the intergenic distances between adjacent co-directional genes (see Figure 3). All proximons included in MetaProx were obtained using a minimum threshold of confidence that is equivalent to a positive predictive value of 0.90: In other words, 90% of the proximons are expected to represent true metagenomic operons based on evidence from known operons of *Escherichia coli* K12 contained in RegulonDB (7). However, it is important to point out that the accuracy of any predicted proximon is contingent on the corresponding accuracy of the coordinates of its member genes and metagenomic gene prediction represents an inherently challenging task. For example, metagenomic gene prediction can be effected by the ability to correctly assemble metagenomic sequence reads into longer contigs, and this process can be subsequently impacted by factors such as sequencing coverage and chimerism (14).

**Figure 4.**
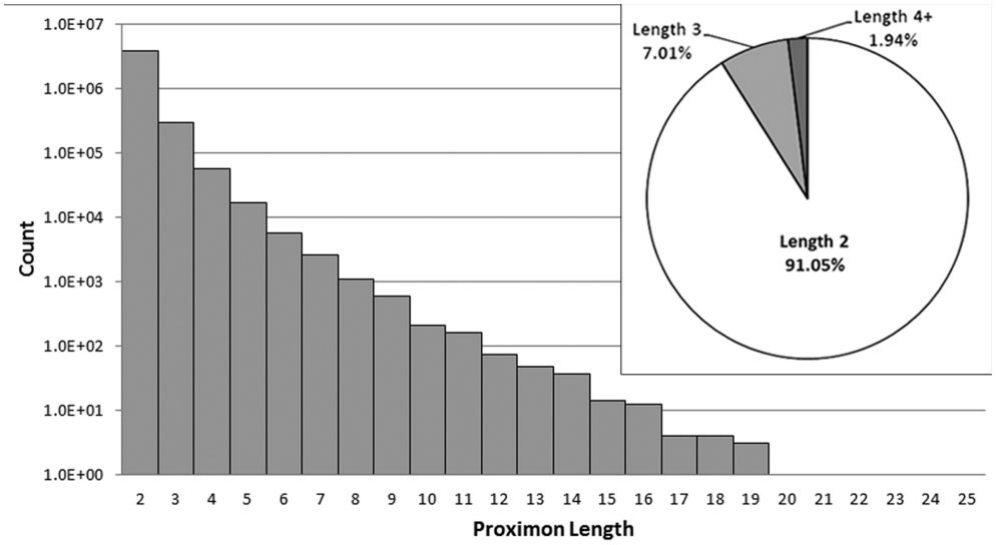
Distribution of proximon lengths. The main panel shows the distribution of proximon lengths with respect to frequency of occurrence using a log (base 10) scale. The inset shows the relative proportion (%) of binary proximons, ternary proximons and proximons with lengths greater than three member genes.

MetaProx currently consists of 4 210 818 proximon objects, and all data are categorized according to the taxonomic system used by the IMG/M (see Table 2). Proximon lengths ranged from 2 to 25 member genes with no proximons of length 22 or 23. Given that the complete set of proximons is composed of 8 926 993 member genes, the vast majority of proximons are binary proximons (i.e. consist of two member genes) with only 9% of all proximons containing more than two member genes (see Figure 4).

**Table 2:** MetaProx database composition

Ecosystem	Count	%	Category	Count	%
Engineered	246 919	5.9%	Bioremediation	48 111	1.1%
			Biotransformation	94 339	2.2%
			Solid waste	65 156	1.5%
			Wastewater	39 313	0.9%
Environmental	3 188 109	75.7%	Air	6647	0.2%
			Aquatic	2 258 143	53.6%
			Terrestrial	923 319	21.9%
Host-associated	775 790	18.4%	Arthropoda	395 549	9.4%
			Birds	63 329	1.5%
			Human	5075	0.1%
			Mammals	150 050	3.6%
			Microbial	5183	0.1%
			Mollusca	27 761	0.7%
			Plants	128 843	3.1%

The database composition is shown according to proximon count and proportion (% of total count) versus metagenomic ecosystem and also for the categories within each respective ecosystem. The total for the category percentages within an ecosystem can differ from the ecosystem percentage due to rounding of the individual category values.

## Deployment

**Figure 5.**
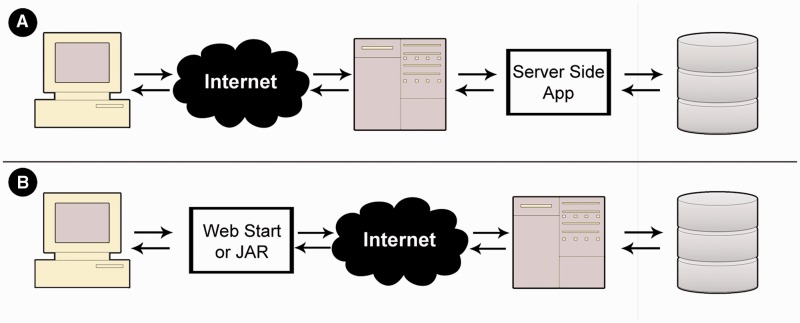
Application deployment perspectives. (**A**) In a typical deployment scenario, a web interface is used to invoke a server-side application that subsequently queries a backend database. (**B**) In contrast, MetaProx deployment provides a client-side JAR or Java Web Start application that directly interacts with a server-side database.

MetaProx is deployed using a distributed client-server model. Commonly, client-server interaction involves a client-side web interface that is used to request server-side processing that often involves subsequent retrieval from a backend database (15) (see Figure 5). MetaProx, however, uses a distribution where the client owns the application (i.e. the search tool) that in turn invokes the server solely for access to the database (see Figure 5). Specifically, the MetaProx database responds to client requests by sending indexed blocks of proximon objects, thereby minimizing physical I/O while emulating a logical perspective where all data are readable by any given application instance (16). The received blocks are subsequently subjected to additional query criteria that are carried out by the client’s unique application instance, running on their own local machine. The benefit of this distributed approach is that clients provide many of their own resources (e.g. memory and CPU), therefore allowing them to take advantage of their own hardware capabilities while simultaneously alleviating the limitations of server-imposed quotas. For example, the maximum number of proximon objects that can be returned by any given search is greatly affected by the amount of memory that the client has elected to allocate for the Java virtual machine. Using modest hardware, we have benchmarked the performance of the search tool and have determined the search rate to be roughly 2400 proximon objects per second, although the rate at any given time can be highly variable (see Supplementary Data).

**Figure 6.**
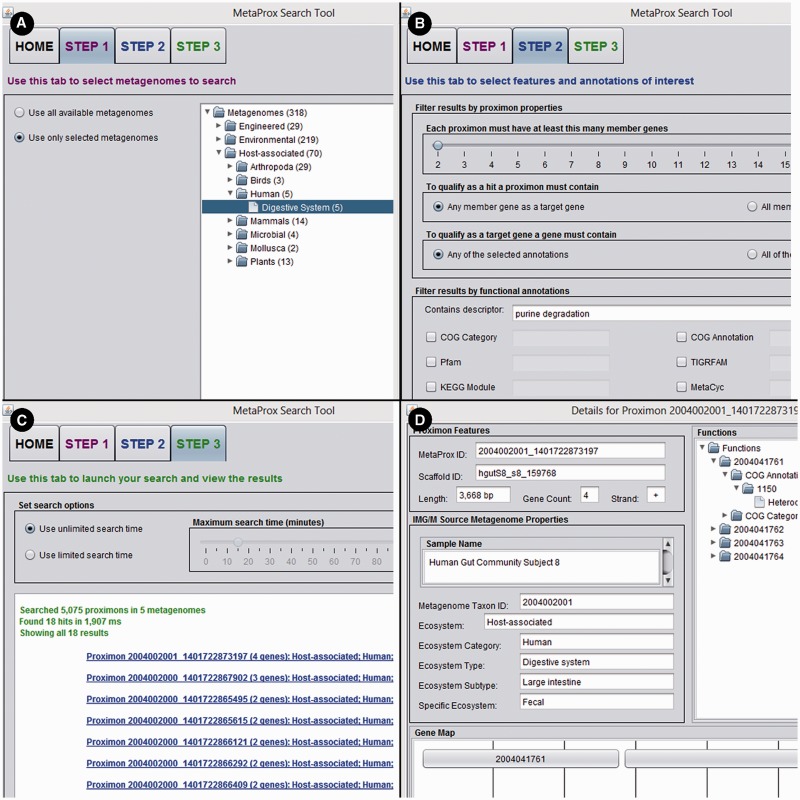
MetaProx graphical user interface. Portions of the MetaProx graphical user interface are shown including the Source tab (**A**), the Target tab (**B**) and the Query tab (**C**). Clicking on a proximon link in the Query tab will display the corresponding Proximon Details panel (**D**) and clicking on a gene link in the Proximon Details panel will display the corresponding Gene Details panel (not shown).

The MetaProx search tool is deployed as a Java ARchive (JAR) that can be either downloaded from the website or launched directly from the browser using Java Web Start technology (17). Although the JAR is identical for both search modes, using a local downloaded JAR can typically circumvent the permissions and security issues that can arise from Java Web Start launches. In either case, the JAR will run a GUI application on the client machine that provides a simple stepwise search protocol (see Figure 6). Search results can be saved using the MetaProx serialized object format or alternatively saved as delimited text for further processing with other tools and pipelines. It is also possible to extract various annotation categories to expedite the construction of metagenomic annotation networks (5) (see Applications).

## Applications

MetaProx has been designed to facilitate the retrieval of metagenomic functional annotations. For example, a user might want to gain insight about cellulase genes from soil metagenomes. The corresponding MetaProx search would provide proximons that meet these constraints and reveal information about the targeted genes but also about the genes that are potentially interacting with the targets. Furthermore, MetaProx offers features to save the retrieved proximon data and also to extract specific functional annotations for easy construction of metagenomic annotation networks using network analysis software, such as Cytoscape (18).

**Figure 7.**
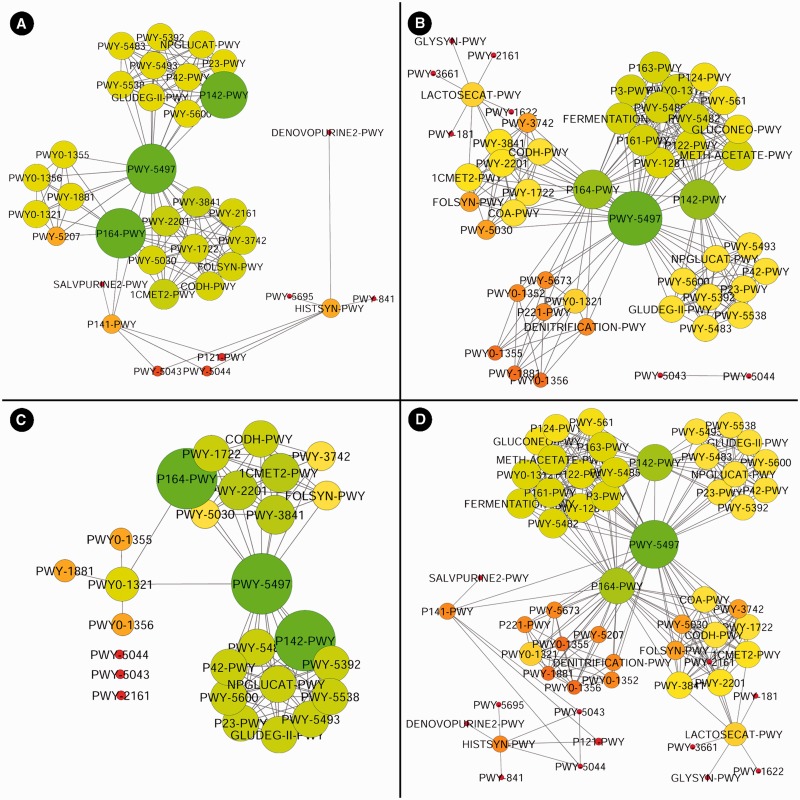
Purine degradation networks. Purine degradation networks are shown for MetaCyc pathways from human digestive metagenomes (**A**), soil metagenomes (**B**), their intersection (**C**) and their union (**D**) where node diameter and brightness (greenness) increase with increasing edge count.

Here, we provide a working example using the MetaProx search tool where purine degradation genes are contrasted from a network perspective using human digestive system metagenomes versus soil metagenomes. First, the source metagenomes are selected from the metagenome tree in Step 1: *Host-associated* → *Human* → *Digestive System* (see Figure 6). Next, the target genes are constrained by entering the keyword ‘purine degradation’ in the descriptor textbox in Step 2 (see Figure 6). Executing this search (Step 3) will return 18 qualifying proximons composed of 39 member genes (see Figure 6). Using the *Save* command followed by the *Save Annotations Only* option allows functional annotations to be saved according to common annotation categories, such as COG (19), Pfam (20), TIGRFAM (21), MetaCyc (22), etc. Here we have selected the MetaCyc pathways and used the annotations to construct a metagenomic annotation network using Cytoscape 2.8.2, and the resulting network contains 35 nodes and 142 edges (see Figure 7). The previous search is repeated but new source metagenomes are selected from the metagenome tree in Step 1: *Environmental* → *Terrestrial* → *Soil*. The 44 qualifying proximons provide MetaCyc pathways that produce a network with 50 nodes and 254 edges (see Figure 7). These networks can be subsequently contrasted, and their intersection (27 nodes and 99 edges) and union (58 nodes and 297 edges) are depicted in Figure 7. This example demonstrates the ease of producing novel functional interaction networks, and we estimate that a novice user could have accomplished this task in roughly half an hour, while an experienced user could have completed it in just a few minutes. The resulting interaction network can then lead to hypothesis generation and experimental validation.

## Future directions

Future directions for MetaProx include increasing the number of proximons contained in the database and expanding the functionality of the search and visualization tools according to the user feedback. We also plan to increase the number of available search settings and to incorporate additional result-filtering options. Query optimization for serialized objects will also be a key focus of future development with the goal of reducing database search times. Similarly, a database block caching policy will also be considered.

To promote the exchange of serialized data, we advocate the establishment of a Biological Object Exchange specification that emulates other existing standards like CORBA. The primary goal of such standardization would be the collaborative creation of an object-oriented specification to define data structures (i.e. classes) that correspond to conventional biological entities, such as genes and proteins. Moreover, the intrinsic property of encapsulation could be applied to combine fundamental structures into diverse composite objects that can easily represent the entire *omics* data sets. Beyond specifying structured data, objects (i.e. specific instances of a given class) also provide methods to interact with the data they contain. This is a pivotal departure from other storage formats because objects simultaneously provide an informational component (via properties), as do other formats, but also a manipulation component (via methods) that is unattainable for textual and markup-based representations. As a result, methods within each class would facilitate the direct manipulation of biological data without the need for tedious file parsing.

Although MetaProx uses an uncommon deployment strategy and advocates a *Data as Data* policy for the representation and dissemination of biological data, the present implementation is a simplified model, and much work remains to be done in the area of serialized data exchange. Enormous strides will be required to emulate the advanced functionality of rigorously tested relational database technologies. However, we are optimistic that the advantages offered by serialized representations of biological data will compel increased interest and research in this topic.

## Supplementary data

Supplementary data are available at *Database* Online.

Supplementary Data
