# Supplementary material for: MetaProx: the database of metagenomic proximons
Source: Database (Oxford). 2014 Oct 4;2014:bau097. doi: 10.1093/database/bau097 (PMC4186327; doi:10.1093/database/bau097)
Supplement: Supplementary Data [file supp_2014_bau097_index.html]

MetaProx: the database of metagenomic proximons — Supplementary Data 

# MetaProx: the database of metagenomic proximons

## Supplementary Data

files

**Files in this Data Supplement:**

- Supplementary Data - xlsx file
